# A furin inhibitor downregulates osteosarcoma cell migration by downregulating the expression levels of MT1-MMP via the Wnt signaling pathway

**DOI:** 10.3892/ol.2014.1839

**Published:** 2014-01-29

**Authors:** BINGSHAN LIU, GUOJUN LI, XIAO WANG, YANG LIU

**Affiliations:** Department of Orthopaedics, Huaihe Hospital, Henan University, Kaifeng, Henan 475001, P.R. China

**Keywords:** α1-PDX, osteosarcoma MG-63 and Saos-2, migration, MT1-MMP, Wnt, β-catenin

## Abstract

This study aimed to explore the exact mechanism of the effect of a furin inhibitor on the migration and invasion of MG-63 and Saos-2 osteosarcoma cells. MG-63 and Saos-2 osteosarcoma cells were treated with regular culture medium in the presence or absence of 480 nM α1-antitrypsin Portland (α1-PDX). Wound-healing and Transwell assays were used for the detection of the effects of α1-PDX on MG-63 and Saos-2 osteosarcoma cell migration and invasion. Western blot analysis and reverse transcription-polymerase chain reaction were performed to detect the expression levels of membrane type I matrix metalloproteinase (MT1-MMP), Wnt and β-catenin. A chromatin immunoprecipitation assay was used for detection of the levels of MT1-MMP gene transcription activity. The results showed that α1-PDX treatment significantly reduced the migration and invasion ability of the cells. Notably, the expression levels of MT1-MMP decreased evidently upon α1-PDX treatment, paralleled with reductions in the expression levels of Wnt and β-catenin. Further analysis of the transcriptional activity of MT1-MMP revealed that the α1-PDX-induced downregulation of the levels of MT1-MMP was mediated by the Wnt signaling pathway. These data suggest that α1-PDX plays a vital role in inhibiting MG-63 and Saos-2 osteosarcoma cell migration and invasion by downregulating the expression levels of MT1-MMP via the Wnt signaling pathway.

## Introduction

Furin is the best-characterized representative of the mammalian subtilisin-like family of proprotein convertases. It is synthesized as an inactive proenzyme and is rapidly matured by autocatalytic cleavage between the prodomain and the catalytic domain in the endoplasmic reticulum (ER) (1,2). Following this initial cleavage, the propeptide-furin complex leaves the ER and enters the trans-Golgi network (TGN) for its second cleavage (3,4). Thus, furin becomes active to process substrate molecules in multiple compartments in the TGN/endosomal system (5). Numerous protein precursors, including matrix metalloproteinases (MMPs), hormones, growth factors, serum proteins, receptors and adhesion molecules, have been identified as furin substrates (6–8). Membrane type I (MT1)-MMP proenzyme cleavage by furin is considered to be a principal event in the activation of this substrate and may play a vital role in tumor cell migration (9).

Furin activation plays a vital role in tumor development (10). The furin inhibitor α1-antitrypsin Portland (α1-PDX) has been used to block furin activity and to prevent cancer metastasis in biochemical, cellular and animal studies (11).

The Wnt signaling pathway plays a vital role in normal development, but also in tumorigenesis (12,13). Inappropriate activation of the Wnt signaling pathway results in the onset of several types of cancer (14). Based on the different interactions between Wnt receptors or co-receptors, the Wnt signaling pathway is divided into three signaling pathways, namely the canonical Wnt/β-catenin signaling pathway, and the non-canonical (or heretical) Wnt/ Ca^2+^ and planar cell polarity (PCP) signaling pathways (15). Previous studies have suggested that the interaction of the Wnt/PCP signaling pathway with certain key molecules is associated with cancer cell migration and invasion (16,17). The canonical Wnt signaling pathway involves a key mediator, β-catenin, which is able to enter the cell nucleus and associate with the transcription factors lymphoid enhancer-binding factor 1 and T-cell factor, leading to the transcription of Wnt target genes (18,19). The stabilization of β-catenin is regulated by phosphorylation modification by glycogen synthase kinase 3β, followed by degradation via the proteasome. Abnormal activation of the Wnt/β-catenin signaling pathway has been detected in a number of types of human tumor, including lung, breast, cervical and liver, and is due to lack of degradation and ultimately the nuclear accumulation of β-catenin. In patients with hepatocellular carcinoma, β-catenin accumulation has been linked to poor differentiation and high proliferative activity of cells, and a poor prognosis (20,21). The levels of β-catenin are regulated by numerous types of protein, which may lead to the onset of cancer if not regulated or expressed appropriately. By forming a complex with transcription factor 4, β-catenin activates the transcription of target genes, including MT1-MMP, whose expression levels correlate with the levels of cell migration and invasion (22). Abnormal expression of MT1-MMP has been detected in numerous types of cancer. Such induction of the expression of MT1-MMP could be regulated by the Wnt/β-catenin signaling pathway; this is based on the observation that depletion of β-catenin in SW480 colorectal carcinoma cells results in the downregulation of the expression levels of MT1-MMP (23). In a previous study, it was demonstrated that the migration of MG-63 and Saos-2 osteosarcoma cells was inhibited significantly by a certain range of concentrations of α1-PDX treatment (10), but the exact molecular mechanism of this effect remains unknown.

## Materials and methods

### Cell culture and experimental reagents

MG-63 and Saos-2 osteosarcoma cells were purchased from the American Type Culture Collection (Manassas, VA, USA) cultured in RPMI-1640 (Invitrogen Life Technologies, Carlsbad, CA, USA) supplemented with 10% fetal bovine serum (FBS), 100 U/ml penicillin and 100 μg/ml streptomycin, in a 5% CO_2_ humidified atmosphere at 37°C. α1-PDX (126850-2.5MG; Calbiochem-Merck KGaA, Darmstadt, Germany) was added to the medium at a concentration of 480 nM where indicated. Primary antibodies against Wnt, β-catenin, MT1-MMP and β-actin were purchased from Santa Cruz Biotechnology, Inc. (Santa Cruz, CA, USA). Other reagents were used, including anti-mouse-IgG-HRP and anti-rabbit-IgG-HRP (BD Biosciences, CA, USA) and Transwell invasion chambers (Promega, Madison, WI, USA).

### Monolayer cell migration assay

A monolayer wound-healing model was performed as a cell migration assay. MG-63 and Saos-2 osteosarcoma cells were seeded in a six-well-plate for 48 h in complete RPMI-1640 medium. A confluent monolayer of MG-63 and Saos-2 osteosarcoma cells was then scraped with a sterile 200-μl pipette tip into another six-well-plate and washed with phosphate-buffered saline (PBS). Following incubation with complete RPMI-1640 or α1-PDX (480 nM) for 48 h, cell migration images were captured using an inverted phase contrast microscope at ×100 magnification.

### Transwell invasion assay

The Matrigel invasion chambers were hydrated for 4 h prior to starting the invasion assay. Log-phase cells (4×10^4^) were plated in 200 μl complete RPMI-1640 containing 10% FBS in the upper chamber of the Transwell, and the lower chamber was filled with 500 μl complete RPMI-1640 containing 10% FBS. Following incubation for 2 h, the cells were treated with α1-PDX as decribed previously and allowed to migrate for 10 h at 37°C and 5% CO_2_. The cells were fixed for 15 min at room temperature by replacing the culture medium in the bottom and top of the chamber with 4% formaldehyde buffer. Subsequently, the chambers were rinsed in PBS and stained with 0.1% crystal violet for 10 min, then the migrated cells were photographed under an optical microscope. The cell number was counted at 12 different areas. Data were averaged from three parallel experiments, which were normalized to those of the controls.

### Reverse transcription-polymerase chain reaction (RT-PCR) analysis

The cells were incubated with α1-PDX for 48 h prior to RT-PCR. Total RNA was extracted from the MG-63 and Saos-2 osteosarcoma cells using the TRIzol method (Invitrogen Life Technologies). RT was performed with 1 μg total RNA and 10 μM of the specific primers. The cDNAs were amplified by PCR for MT1-MMP (sense, 5′-AGCCCCGAAGCCTGGCTACA-3′; and antisense, 5′-GCCGCCCTCACCATCGAAGG-3′; 492-bp product), or glyceraldehyde 3-phosphate dehydrogenase was used as the endogenous reference housekeeping gene (sense, 5′-ACCACAGTCCATGCCATCAC-3′; and antisense, 5′-TCCACCACCCTGTTGCTGTA-3′; 556-bp product). The PCR conditions were as follows: 95°C for 5 min, followed by 30 cycles of 95°C for 15 sec, 60°C for 30 sec and 72°C for 45 sec.

### Western blot analysis

The cells were incubated with α1-PDX for 48 h prior to western blotting. MG-63 and Saos-2 osteosarcoma cells were lysed in radioimmunoprecipitation assay (RIPA) buffer (50 mM Tris pH 7.4, 150 mM NaCl, 1% Triton X-100, 0.1% SDS, 1% sodium deoxycholate, 5 mM EDTA, 100 mM NaF, and 1 mM Na_3_VO_4_) containing a protease inhibitor cocktail (product no., 04693116001; Roche, Madison, WI, USA)for 30 min on ice, followed by centrifugation for 30 min at 35,800 × g. The protein concentrations were determined by the bicinchoninic acid assay method (Pierce BCA Protein Assay kit; Pierce Biotechnology, Inc., Rockford, IL, USA). Equal quantities of total proteins were electrophoresed by 12% SDS-PAGE gel, followed by transfer to polyvinylindene difluoride membranes using a wet transblot system (Bio-Rad, Hercules, CA, USA). The membranes were blocked for 1 h at room temperature with 5% nonfat dry milk and incubated overnight at 4°C with antibodies [rabbit anti-Wnt, β-catenin, MT1-MMP and mouse anti-β-actin (1:1,000)]. After washing, the membranes were incubated for 1 h with HRP-conjugated goat anti-rabbit or anti-mouse-IgG-HRP secondary antibodies diluted to 1:5,000 in PBS Tween-20. After further washing and processing using SuperSignal West Pico Chemiluminescent substrate (Pierce Biotechnology, Inc.), the membranes were exposed to a Fujifilm LAS-3000 Imager (Fuji, Tokyo, Japan). The band densities of the western blots were normalized relative to the relevant β-actin band density with ImageJ Analysis software (National Institutes of Health, Bethesda, MD, USA).

### Chromatin immunoprecipitation (ChIP) assay

The cells were incubated with α1-PDX for 48 h prior to performing the ChIP assay. A ChIP assay was performed using a ChIP kit (Sigma-Aldrich, St. Louis, MO, USA) with slight modifications. MG-63 and Saos-2 osteosarcoma cells (2×10^7^) were cross-linked with 1% formaldehyde for 10 min at room temperature, followed by the addition of 1 ml of 125 mM glycine to inactivate the formaldehyde. The cells were washed twice with ice-cold PBS and then scraped and centrifuged at 1,000 × g at 4°C for 5 min. The pelleted cells were lysed with 1 ml modified-RIPA lysis buffer (0.1% SDS, 10 mM EDTA, 1% Triton X-100 and 50 mM Tris-HCl pH 8.1) containing a protease inhibitor cocktail and incubated on ice for 10 min. Following sonication to produce genomic DNA with lengths of 0.2–0.5 kb, the samples were centrifuged at 13,000 × g for 10 min to remove insoluble cell debris. The lysates were diluted in ChIP dilution buffer (0.01% SDS; 1.1% Triton X-100; 2 mM EDTA; 20 mM Tris-HCl, pH 8.1; and 500 mM NaCl) and protease inhibitor cocktail. Dilutions of the chromatin preparations were stored at −20°C. The chromatin solution was precleared with 20 μl of 3% bovine serum albumin/protein A agarose beads for 2 h at 4°C with rotation. Anti-β-catenin polyclonal antibody (Santa Cruz Biotechnology, Inc.) was added to the precleared supernatant and incubated overnight at 4°C with rotation. The negative controls included a sample incubated without antibody and one incubated with rabbit IgG (Santa Cruz Biotechnology, Inc.) to determine whether the interactions were due to nonspecific IgG interactions. The bead complexes were washed with low-salt immune complex wash buffer (Sigma-Aldrich), followed by high-salt immune complex wash buffer (Sigma-Aldrich) and a final LiCl immune complex wash buffer (Sigma-Aldrich) for 5 min each on a rotating platform followed by brief centrifugation at 35,800 × g for 10 min. Two final washes in 1X Tris EDTA buffer were performed for 5 min each. Following the final wash, the DNA was extracted by incubating the beads twice for 15 min with 200 μl freshly prepared elution buffer (1% SDS and 50 mM NaHCO_3_). The samples were then uncrosslinked in a 65°C water bath overnight and the DNA was purified using a QIAquick Nucleotide Removal kit (Qiagen Inc., Valencia, CA, USA). The purified DNA was analyzed by PCR. The PCR primers used to amplify the MT1-MMP promoter region were as follows: GTCTCCCGCCCCAAGACCCT (forward) and GGAACACCACATCGGGGGCG (reverse).

### Statistical analysis

All experiments were performed three times and the data were expressed as the mean ± standard error of the mean. Statistical analysis was performed by SPSS software, version 11.0 (SPSS, Inc., Chicago, IL, USA). Differences between the groups were statistically evaluated using the t-test or one-way analysis of variance with post-hoc analysis. P<0.05 was considered to indicate a statistically significant difference.

## Results

### Inhibitory effect of α1-PDX on MG-63 and Saos-2 osteosarcoma cell migration

**Figure 1:**
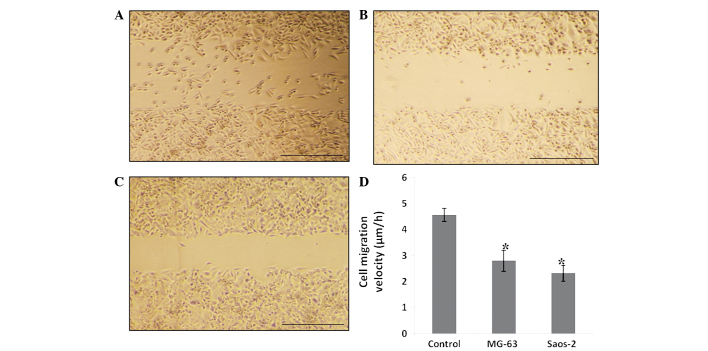
Regulation of cell migration by α1-PDX in osteosarcoma MG-63 and Saos-2 cells. (A) MG-63 log-phase cells treated with normal complete RPMI-1640, and (B) MG-63 and (C) Saos-2 osteosarcoma cells treated with 480 nM α1-PDX. Monolayer cells were scraped with a 200-μl sterile tip. Images of cell migration were captured with an inverted phase contrast microcope (magnification, ×20)and the migration distance was used for statistical analysis of (D) the cell migration velocity. Data were expressed as the mean cell migration velocity ± SEM from three separate experiments. Statistical analyses were performed using the t-test and one-way analysis of variance. ^*^P<0.05 indicates a significant difference compared with the control group. α1-PDX, α1-antitrypsin Portland.

**Figure 2:**
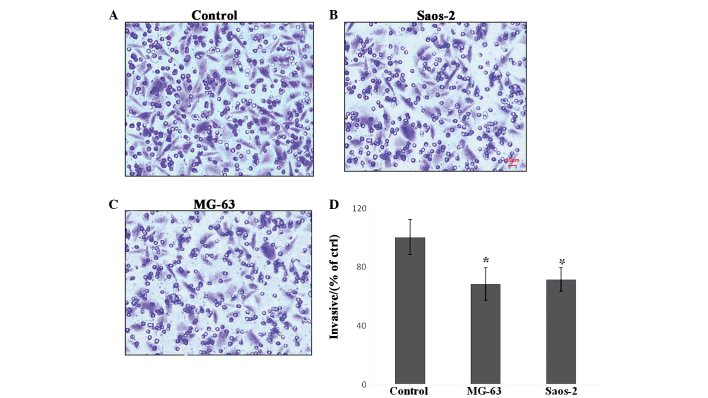
Regulation of invasion by α1-PDX in MG-63 and Saos-2 osteosarcoma cells. The Transwell system was used for detection of the invasion ability of osteosarcoma cells upon α1-PDX treatment. (A) MG-63 control cells. (B) Saos-2 and (C) MG-63 osteosarcoma cells were treated with complete RPMI-1640 or 480 nM α1-PDX, seeded in the upper chamber at a density of 4×10^4^ cells per chamber and allowed to migrate through the chamber pore for 12 h. Subsequently, the number of cells per field that had migrated to the bottom surface of the filter was counted. (D) The mean and standard error from three separate experiments are shown. Statistical analyses were performed using the t-test and one-way analysis of variance. ^*^P<0.05 indicates a significant difference compared with the control group. α1-PDX, α1-antitrypsin Portland.

The effect of α1-PDX on MG-63 and Saos-2 osteosarcoma cell migration was monitored by a monolayer wound-healing assay. Log-phase cells were seeded on six-well plates and incubated with complete cell medium or 480 nM α1-PDX for 24 h. Following wounding by a sterile 200-μl pipette tip, the cells treated with normal cell medium migrated clearly. The cells that were treated with α1-PDX showed no evident migration (Fig. 1). In the three-dimensional cell migration assay with the Transwell system, the cells treated with α1-PDX were found to migrate less than the control cells (Fig. 2). This data indicates that MG-63 and Saos-2 osteosarcoma cell migration was inhibited upon α1-PDX treatment. However, these assays did not reveal the mechanism of the inhibitory effect.

### Downregulation effect of α1-PDX on the expression levels of MT1-MMP

**Figure 3:**
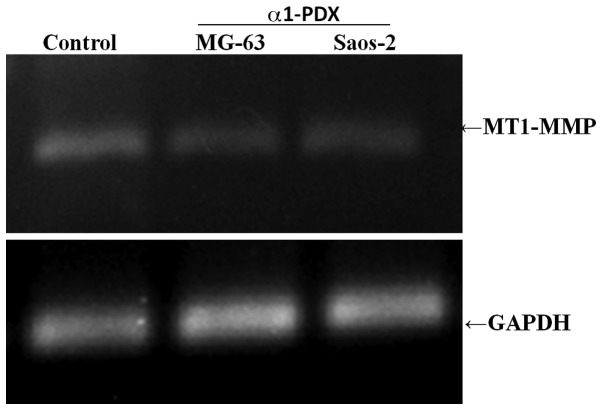
Effect of α1-PDX on the mRNA expression levels of MT1-MMP in MG-63 and Saos-2 osteosarcoma cells. The cells were treated with completed cell culture medium or 480 nM α1-PDX for 48 h and the mRNA of MT1-MMP was analyzed by reverse transcription-polymerase chain reaction. The mRNA of GAPDH was used for the internal control, which indicated the equal total of mRNA. α1-PDX, α1-antitrypsin Portland; MT1-MMP, membrane type 1-matrix metalloproteinase; GAPDH, glyceraldehyde 3-phosphate dehydrogenase.

**Figure 4:**
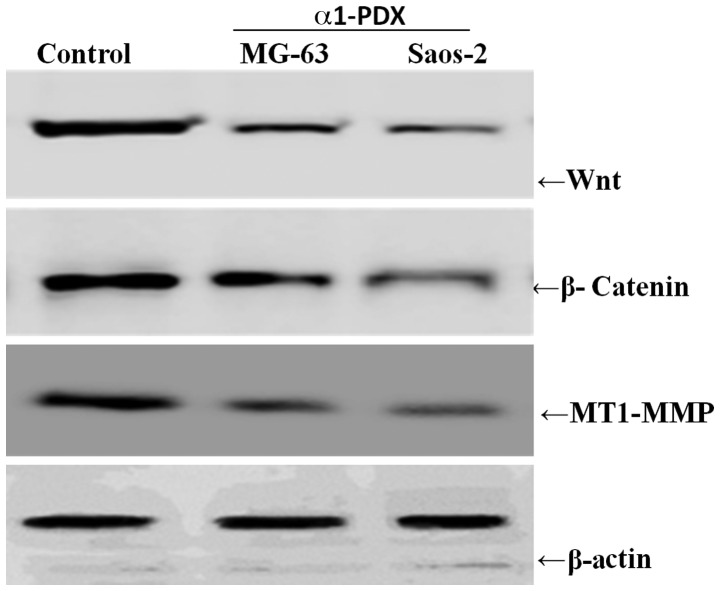
Effect of α1-PDX on the expression levels of MT1-MMP, Wnt and β-catenin in MG-63 and Saos-2 osteosarcoma cells. The total proteins were extracted from the cells and the expression levels of Wnt, β-catenin and MT1-MMP were detected with western blot analysis. Equal loading protein was shown with the β-actin immunoblot. α1-PDX, α1-antitrypsin Portland; MT1-MMP, membrane type 1-matrix metalloproteinase.

To explore the possible mechanism of the inhibitory effect of α1-PDX on MG-63 and Saos-2 osteosarcoma cell migration, the expression levels of MT1-MMP, which is the key mediator of cell migration and invasion, were detected. RT-PCR was used for detection of the levels of gene expression of MT1-MMP in MG-63 and Saos-2 osteosarcoma cells upon α1-PDX treatment. From the results (Fig. 3), the gene expression levels of MT1-MMP were reduced significantly in the α1-PDX treatment cells, compared with those of the control group. The protein levels of MT1-MMP were also detected upon α1-PDX treatment and, as expected, the protein levels also decreased evidently compared with those of the control cells (Fig. 4). These results suggest that α1-PDX suppresses MG-63 and Saos-2 osteosarcoma cell migration, possibly via downregulation of the expression of MT1-MMP gene and protein levels.

### Wnt signaling pathway may be involved in the downregulation of the levels of MT1-MMP by α1-PDX treatment

To further explore the exact mechanism by which α1-PDX downregulates the MT1-MMP expression levels, the effect of α1-PDX on Wnt signaling pathway-related properties was investigated. When the MG-63 and Saos-2 osteosarcoma cells were treated with α1-PDX for the indicated times, the expression levels of Wnt and β-catenin decreased significantly, paralleled with the reduction in the MT1-MMP expression levels (Fig. 4). These results suggested that the downregulating effect of α1-PDX on the levels of MT-MMP may be via the Wnt signaling pathway.

### Effect of α1-PDX on the levels of MT1-MMP transcriptional activity via the Wnt signaling pathway

**Figure 5:**
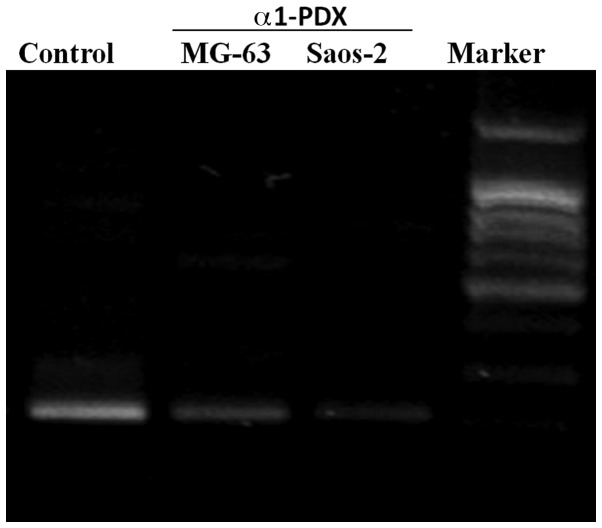
Regulation of α1-PDX on the levels of transcriptional activity of MT1-MMP in osteosarcoma MG-63 and Saos-2 cells. The cells were treated as indicated; whole cell lysates were sonicated to produce genome DNA and chromatin samples were immunoprecipitated with β-catenin antibody, and normal rabbit IgG, then MT1-MMP was amplified by 35 cycles of polymerase chain reaction. α1-PDX, α1-antitrypsin Portland; MT1-MMP, membrane type 1-matrix metalloproteinase.

To determine whether the levels of MT1-MMP transcriptional activity correlated with the levels of Wnt signaling pathway activity, a ChIP assay was performed. The MG-63 and Saos-2 osteosarcoma cells were treated with α1-PDX for 24 h and β-catenin antibody was used for immunoprecipitation with target genes. The results showed that the levels of MT1-MMP transcriptional activity decreased evidently compared with those of the control (Fig. 5), which demonstrated that the effect of α1-PDX on MT1-MMP expression levels was mediated through Wnt signaling.

## Discussion

Previous studies have indicated that α1-PDX has the potential role of inhibiting cancer cell migration, but the exact mechanism is unknown. In the present study, it has been demonstrated that α1-PDX inhibited the migration and invasion of MG-63 and Saos-2 osteosarcoma cells through downregulation of the expression levels of MT1-MMP via the Wnt/β-catenin signaling pathway. The invasion ability of osteosarcoma MG-63 and Saos-2 cells upon α1-PDX treatment was detected through two- and three-dimensional migration assays (wound-healing and Transwell assays). It was observed that the migration ability was reduced significantly upon α1-PDX treatment for 24 h. Tumor invasion and metastasis is a multistage and multifactorial process, which is regulated by complicated mechanisms and multiple signaling pathways (24).

Numerous protein molecules are involved in the regulation of cell adhesion, migration and invasion in tumor biology behaviors. MMPs are a family of zinc-binding proteases that have been shown to contribute to cancer cell invasion through the ability to degrade the extracellular matrix (25,26). MT1-MMP (also known as MMP-14) is the first identified and also the most common member of the MT-MMP subfamily involved in pericellular proteolysis associated with cell migration (27,28).

Harada *et al* and Arii *et al* have demonstrated that MT1-MMP is involved in hepatocarcinoma cell migration (29,30); however, the molecular mechanism is unknown. In searching for the underlying mechanism of α1-PDX inhibition of the migration and invasion of MG-63 and Saos-2 osteosarcoma cells in the present study, the expression levels of MT1-MMP mRNA and protein in MG-63 and Saos-2 osteosarcoma cells upon α1-PDX treatment were detected. It was identified that the mRNA and protein expression levels of MT1-MMP were decreased evidently upon α1-PDX treatment.

The activity levels of the majority of MMPs are very low in normal steady state tissues; however, their expression levels are regulated by various inflammatory cytokines, growth factors and hormones, as well as by cell-cell interactions (31). Furthermore, the proteolytic activity of MMPs is strictly controlled at several levels, including transcriptional, post-transcriptional and post-translational, as well as via their endogenous inhibitors (31,32). The transcription of MT1-MMP is strictly regulated by the Wnt signaling pathway (33,34); therefore, we hypothesized that inhibition of MG-63 and Saos-2 osteosarcoma cell migration and invasion by α1-PDX may be through downregulation of the Wnt signaling pathway. Based on this assumption, the expression levels of Wnt and β-catenin were detected in the present study. Western blotting indicated that the expression levels of Wnt and β-catenin decreased markedly in the α1-PDX-treated cells compared with those of the control; however, the expression levels of the positive control, the docetaxel-treated group, decreased weakly. These results suggested that α1-PDX has a potential role of downregulating the expression levels of Wnt and β-catenin. To investigate whether the effect of α1-PDX on the transcriptional activity of MT1-MMP is mediated through the Wnt signaling pathway, the levels of MT1-MMP transcriptional activity were detected through a ChIP assay. As expected, upon α1-PDX treatment a small level of MT1-MMP was detected, which was decreased significantly compared with that of the control.

In summary, the data of the present study demonstrated that α1-PDX has the potential role of inhibiting the migration and invasion of MG-63 and Saos-2 osteosarcoma cells, which may be through downregulating the expression levels of MT1-MMP via the canonical Wnt signaling pathway. It is of note that α1-PDX downregulates the expression levels of transcripts and protein of MT1-MMP, an activator of proMMP-2 (pro-gelatinase A/72 kDa type IV collagenase) (35), which is a major and specific basement membrane matrix protein. Since the degradation of the basement membrane by MMP-2 is likely an essential step for cancer invasion (36–38), it is necessary to study whether α1-PDX mediates the activity of other MMPs. Therefore, numerous aspects of the mechanism of α1-PDX remain to be resolved.

## References

[1] Gawlik K, Shiryaev SA, Zhu W (2009). Autocatalytic activation of the furin zymogen requires removal of the emerging enzyme’s N-terminus from the active site. PLoS One.

[2] Vey M, Schäfer W, Berghöfer S, Klenk HD, Garten W (1994). Maturation of the trans-Golgi network protease furin: compartmentalization of propeptide removal, substrate cleavage, and COOH-terminal truncation. J Cell Biol.

[3] Creemers JW, Vey M, Schäfer W, Ayoubi TA, Roebroek AJ, Klenk HD, Garten W, Van de Ven WJ (1995). Endoproteolytic cleavage of its propeptide is a prerequisite for efficient transport of furin out of the endoplasmic reticulum. J Biol Chem.

[4] Anderson ED, VanSlyke JK, Thulin CD, Jean F, Thomas G (1997). Activation of the furin endoprotease is a multiple-step process: requirements for acidification and internal propeptide cleavage. EMBO J.

[5] Molloy SS, Thomas L, VanSlyke JK (1994). Intracellular trafficking and activation of the furin proprotein convertase: localization to the TGN and recycling from the cell surface. EMBO J.

[6] Fujisawa T, Kamimura H, Hosaka M (2004). Functional localization of proprotein-convertase furin and its substrate TGFbeta in EGF receptor-expressing gastric chief cells. Growth Factors.

[7] Louagie E, Taylor NA, Flamez D (2008). Role of furin in granular acidification in the endocrine pancreas: identification of the V-ATPase subunit Ac45 as a candidate substrate. Proc Natl Acad Sci USA.

[8] Yana I, Weiss SJ (2000). Regulation of membrane type-1 matrix metalloproteinase activation by proprotein convertases. Mol Biol Cell.

[9] Dangi-Garimella S, Krantz SB, Barron MR (2011). Three-dimensional collagen I promotes gemcitabine resistance in pancreatic cancer through MT1-MMP-mediated expression of HMGA2. Cancer Res.

[10] López de Cicco R, Bassi DE, Zucker S (2005). Human carcinoma cell growth and invasiveness is impaired by the propeptide of the ubiquitous proprotein convertase furin. Cancer Res.

[11] Thomas G (2002). Furin at the cutting edge: from protein traffic to embryogenesis and disease. Nat Rev Mol Cell Biol.

[12] Nusse R, Varmus HE (1992). Wnt genes. Cell.

[13] McMahon AP, Gavin BJ, Parr B (1992). The Wnt family of cell signalling molecules in postimplantation development of the mouse. Ciba Found Symp.

[14] Polakis P (2007). The many ways of Wnt in cancer. Curr Opin Genet Dev.

[15] Smalley MJ, Dale TC (1999). Wnt signalling in mammalian development and cancer. Cancer Metastasis Rev.

[16] Wang Y (2009). Wnt/Planar cell polarity signaling: a new paradigm for cancer therapy. Mol Cancer Ther.

[17] Katoh M (2005). WNT/PCP signaling pathway and human cancer (review). Oncol Rep.

[18] Hecht A, Vleminckx K, Stemmler MP (2000). The p300/CBP acetyltransferases function as transcriptional coactivators of beta-catenin in vertebrates. EMBO J.

[19] Takemaru KI, Moon RT (2000). The transcriptional coactivator CBP interacts with beta-catenin to activate gene expression. J Cell Biol.

[20] Inagawa S, Itabashi M, Adachi S (2002). Expression and prognostic roles of beta-catenin in hepatocellular carcinoma: correlation with tumor progression and postoperative survival. Clin Cancer Res.

[21] Wong CM, Fan ST, Ng IO (2001). beta-Catenin mutation and overexpression in hepatocellular carcinoma: clinicopathologic and prognostic significance. Cancer.

[22] Pulyaeva H, Bueno J, Polette M, Birembaut P, Sato H, Seiki M, Thompson EW (1997). MT1-MMP correlates with MMP-2 activation potential seen after epithelial to mesenchymal transition in human breast carcinoma cells. Clin Exp Metastasis.

[23] Takahashi M, Tsunoda T, Seiki M, Nakamura Y, Furukawa Y (2002). Identification of membrane-type matrix metalloproteinase-1 as a target of the beta-catenin/Tcf4 complex in human colorectal cancers. Oncogene.

[24] Kessenbrock K, Plaks V, Werb Z (2010). Matrix metalloproteinases: regulators of the tumor microenvironment. Cell.

[25] Itoh Y, Seiki M (2006). MT1-MMP: a potent modifier of pericellular microenvironment. J Cell Physiol.

[26] Kajita M, Itoh Y, Chiba T, Mori H, Okada A, Kinoh H, Seiki M (2001). Membrane-type 1 matrix metalloproteinase cleaves CD44 and promotes cell migration. J Cell Biol.

[27] Deryugina EI, Ratnikov BI, Postnova TI, Rozanov DV, Strongin AY (2002). Processing of integrin alpha(v) subunit by membrane type 1 matrix metalloproteinase stimulates migration of breast carcinoma cells on vitronectin and enhances tyrosine phosphorylation of focal adhesion kinase. J Biol Chem.

[28] Overall CM (2002). Molecular determinants of metalloproteinase substrate specificity: matrix metalloproteinase substrate binding domains, modules, and exosites. Mol Biotechnol.

[29] Harada T, Arii S, Mise M, Imamura T, Higashitsuji H (1998). Membrane-type matrix metalloproteinase-1(MT1-MMP) gene is overexpressed in highly invasive hepatocellular carcinomas. J Hepatol.

[30] Arii S, Mise M, Harada T, Furutani M, Ishigami S, Niwano M (1996). Overexpression of matrix metalloproteinase-9 gene in hepatocellular carcinoma with invasive potential. Hepatology.

[31] Nagase H, Visse R, Murphy G (2006). Structure and function of matrix metalloproteinases and TIMPs. Cardiovasc Res.

[32] Egeblad M, Werb Z (2002). New functions for the matrix metalloproteinases in cancer progression. Nat Rev Cancer.

[33] Kähäri VM, Saarialho-Kere U (1999). Matrix metalloproteinases and their inhibitors in tumour growth and invasion. Ann Med.

[34] Sato H, Takino T, Okada Y, Cao J, Shinagaw A, Yamamoto E, Seiki M (1994). A matrix metalloproteinase expressed on the surface of invasive tumour cells. Nature.

[35] Sounni NE, Noel A (2005). Membrane type-matrix metalloproteinases and tumor progression. Biochimie.

[36] Sato H, Takino T, Miyamori H (2005). Roles of membrane-type matrix metalloproteinase-1 in tumor invasion and metastasis. Cancer Sci.

[37] Seiki M (2003). Membrane-type 1 matrix metalloproteinase: a key enzyme for tumor invasion. Cancer Lett.

[38] Holmbeck K, Bianco P, Yamada S, Birkedal-Hansen H (2004). MT1-MMP: a tethered collagenase. J Cell Physiol.

